# How early olfactory experiences influence brain development in mice

**DOI:** 10.3389/fncir.2025.1608270

**Published:** 2025-06-10

**Authors:** Hirofumi Nishizumi

**Affiliations:** Department of Brain Function, School of Medical Sciences, University of Fukui, Eiheiji, Fukui, Japan

**Keywords:** sensory systems, activity, olfactory circuit, critical period, plasticity, olfactory imprinting, memory

## Abstract

Mammalian sensory systems develop through both activity-dependent and activity-independent processes. While the foundational neural circuits are encoded by genetics, their refinement depends on activity-driven mechanisms. During the neonatal critical period - a specific developmental phase - sensory circuits adapt and mature in response to environmental stimuli. Initially, this plasticity is reversible, but over time, it becomes permanent. Lack of adequate stimulation during this phase can lead to impaired neural function, highlighting the importance of sensory input for optimal system development. In mice, olfactory neural circuits are first established largely through genetic programming. However, early exposure to environmental odors is crucial in shaping these circuits, affecting both odor perception and social behaviors. This review explores recent findings on the development of olfactory circuits in mice and their impact on behavior.

## Introduction

Experience-dependent neuronal plasticity is a key feature of the nervous system. When the brain is exposed to environmental stimuli, it responds by refining its circuits and increasing plasticity. In the early stages of life, there is a specific time frame when neuronal circuits are especially adaptable to experiences. This period of heightened plasticity is called the critical period. Hubel and Wiesel first described the critical period in 1962 (Hubel and Wiesel, 1962), during their study of how binocular vision develops in the visual cortex. Since then, researchers have found critical periods in various sensory systems across different species (Hensch, 2004; Hensch, 2005; Reha et al., 2020), including in the visual, auditory, somatosensory, and olfactory systems of mice (Jeanmonod et al., 1981; Lieff et al., 1975; Ma et al., 2014; Tsai and Barnea, 2014). In this review, I will explore how experience-dependent plasticity occurs in the mouse olfactory system both during and after the critical period.

## Early life stages of mice

**Figure 1:**
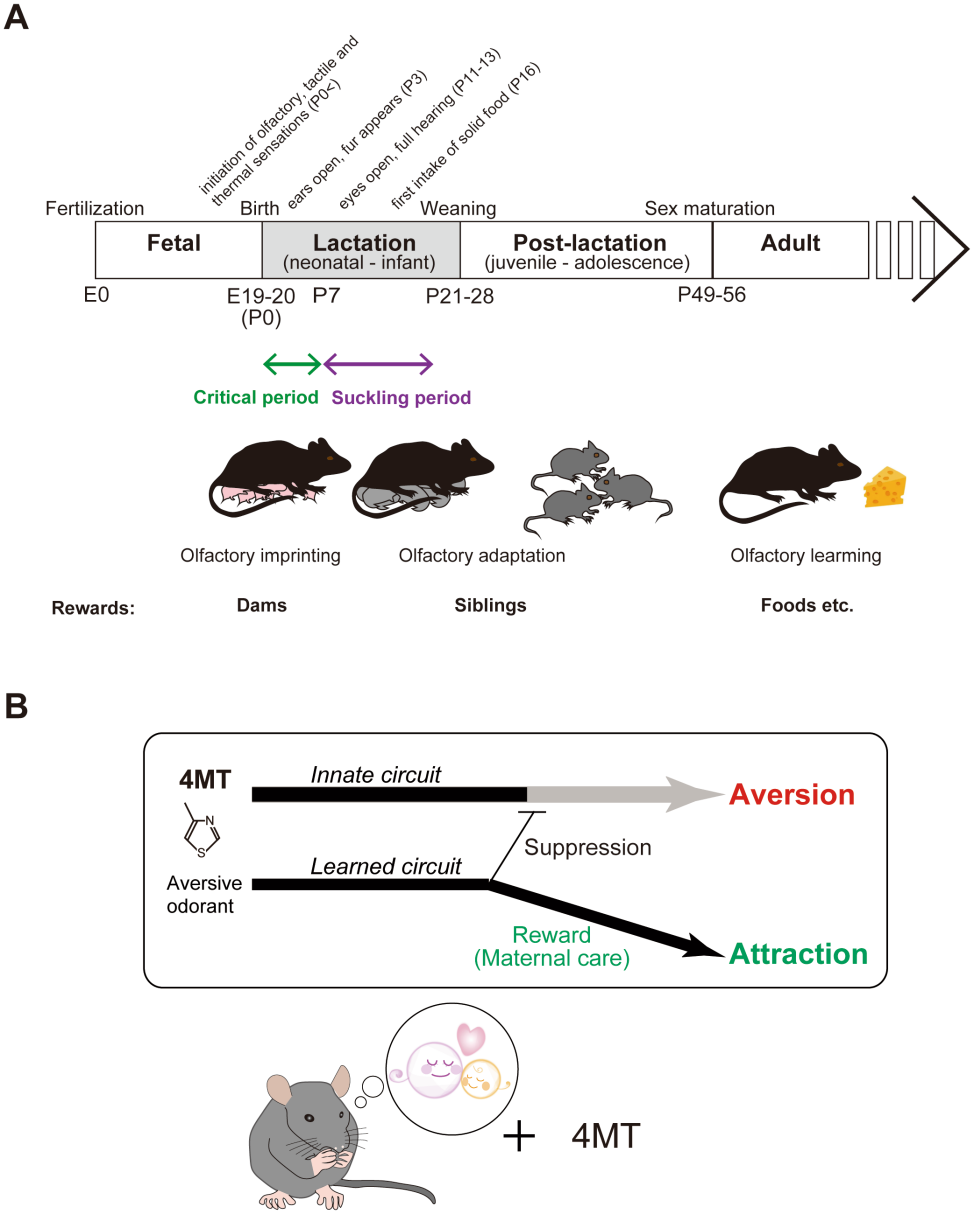
**(A)** Development of physiology and behavior over the lifetime of a mouse. Events are indicated at the mean time point of their occurrence. E, embryonic day; P, postnatal day. **(B)** A schematic diagram of the neural circuitry processing an aversive odorant, 4MT. While 4MT typically induces aversive behavior in mice through instinctive circuits, mouse pups exposed to 4MT during lactation period with their mothers and siblings develop an attractive response to it. In such cases, the neural circuits associating 4MT with a pleasant odor are believed to not only trigger attractive behavior but also suppress the instinctive circuits responsible for aversive responses.

The developmental period of mice can be roughly divided into fetal, lactation (neonatal to infant), post-lactation (juvenile to adolescence), and adult stages (Figure 1A). During the fetal stage, fertilized eggs divide and a variety of cell types, specialized tissues, and organs are shaped. In the late fetal stage, motor-sensory and cognitive behaviors can be observed in rodents (Smotherman and Robinson, 1985; Busnel et al., 1992). Mouse pups undergo significant environmental changes after birth, including temperature and nutritional variability, as well as competition with siblings for resources. They are born in an immature state but possess whiskers to process tactile, olfactory, and thermal cues (Latham and Mason, 2004). Early development includes opening ears around postnatal day 3 (P3) (Theiler, 1972), opening eyes around P11 (Williams and Scott, 1953; Theiler, 1972), first intake of solid food (P16) (Ost'adalova and Babicky, 2012), and gradual sensory and motor improvements such as walking and exploratory behavior. Then, independence begins with weaning around P21-25 (Le Roy et al., 2001), leading to reduced reliance on maternal care and increased behavioral maturation. The post-lactation stage is the developmental transition from childhood to adulthood.

## Olfactory circuit formation in mice

The olfactory system plays a crucial role in the survival of animals. It provides vital cues about the chemical environment that allows an organism to optimize feeding, reproduction, predator avoidance, and social conduct. In mice, volatile odorants are received by odorant receptors (ORs) expressed in the olfactory sensory neurons (OSNs) in the olfactory epithelium (Buck and Axel, 1991). OSNs expressing the same OR project their axons to a set of glomeruli in the olfactory bulb (Mombaerts et al., 1996). Then odor information is converted to a combinatorial pattern of activated glomeruli in the olfactory bulb (Malnic et al., 1999). Incidentally, OSNs begin to target their axons to the olfactory bulb around embryonic day 15 (E15). The initially formed glomeruli are ambiguously shaped, containing a mixed subpopulation of OSN axons. By about P10, however, distinct glomeruli emerge slowly and involve considerable axonal reorganization to achieve the highly topographical projection as observed in adults (Treloar et al., 1999). Odor information from each glomerulus is further transmitted to various higher-order olfactory processing centers by second-order neurons, mitral/tufted (M/T) cells, that ultimately give rise to olfactory perception (Wilson and Sullivan, 2011).

The mouse olfactory system contains two distinct neural pathways, innate and learned, which carry odor signals from the olfactory bulb to the olfactory cortex for perception and decision-making (Figure 1B) (Kobayakawa et al., 2007). For instinctive decisions, odor information is directly sent to specific valence regions in the amygdala (Root et al., 2014; Inokuchi et al., 2017). In contrast, learned decisions involve transmitting olfactory signals to the piriform cortex for odor perception and the recall of associated memories (Wilson and Sullivan, 2011; Russo et al., 2020; Chen et al., 2022; Poo et al., 2022).

During development, innate circuits become active around birth, preceding the activation of learned circuits (Hall and Swithers-Mulvey, 1992). While instinctive decisions are typically fixed, they can be altered through odor experiences (Sullivan et al., 2000; Logan et al., 2012; Qiu et al., 2021). During the critical neonatal period, sensitivity to imprinted odors increases, and positive qualities are added to odor memories (Inoue et al., 2021). Environmental odor stimuli promote the growth of projection-neuron dendrites and the formation of synapses within glomeruli (Liu et al., 2016; Inoue et al., 2018). Qiu et al. (2021) found that prolonged exposure to a specific odor during the critical period alters the innervation pattern of OSNs expressing the corresponding receptor, leading to changes in odor perception later in life. For instance, modifications in projection patterns reduced strong attraction to odors like urine and peanut butter, as well as aversion to the smell of spoiled food. This phenomenon may be analogous to immune system tolerance. Therefore, olfactory perception remains flexible and responsive to environmental odors beyond the neonatal stage (Valle-Leija et al., 2012; Geramita and Urban, 2016; Liu and Urban, 2017).

## Olfactory critical period in mice

The critical period of sensory-driven plasticity in mice is shaped by sensory stimuli. Research into this phenomenon often involves deprivation or excessive exposure to sensory input. Ducklings follow the first moving object after hatching, recognizing it as a parental bird (Lorenz, 1935). Eye mask experiments performed during the critical period, which allow activity-dependent circuit formation to occur, are widely known (Wiesel and Hubel, 1963). Although these experiments were reported decades ago, not so much is known about plastic changes in the sensory system at the molecular level.

**Figure 2:**
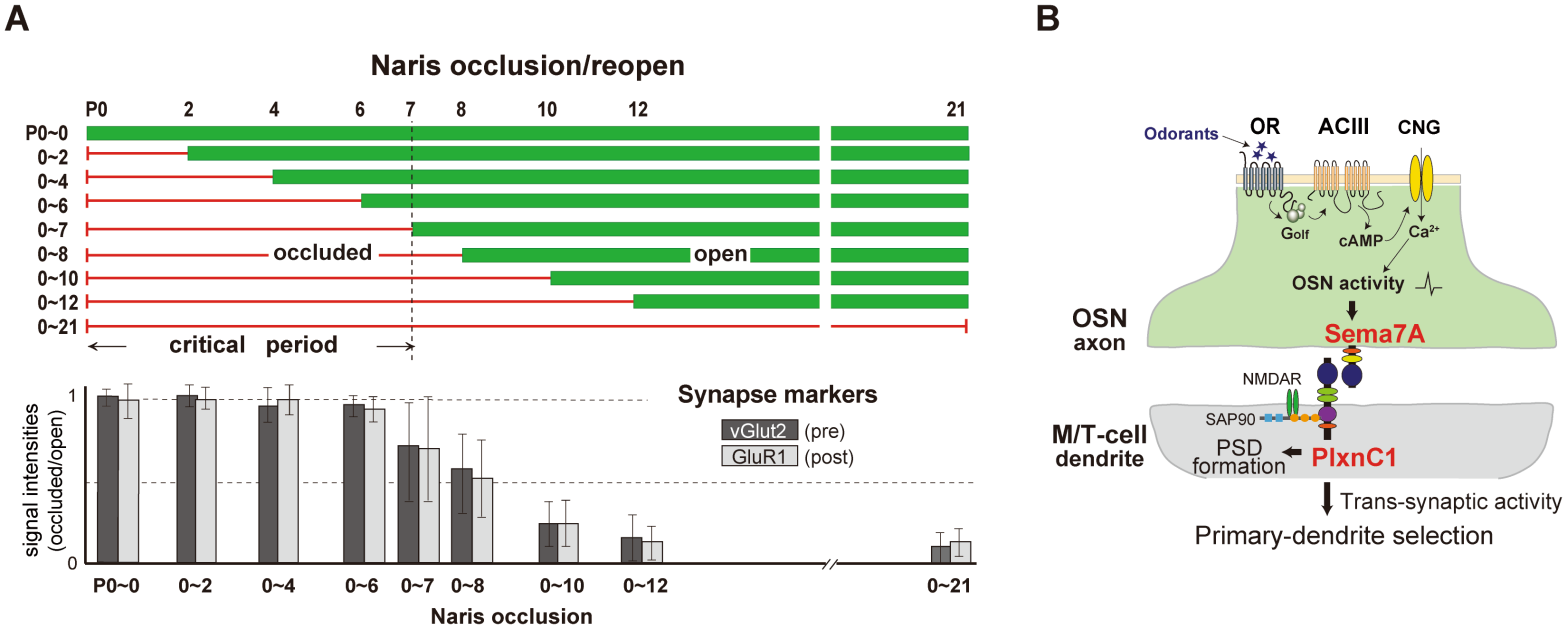
**(A)** Unilateral naris occlusion in neonatal mice. Mice underwent unilateral naris occlusion at P0, with the occluded naris reopened at various time points. OB samples were collected at P21 and analyzed using immunostaining to assess pre-and post-synaptic markers, vGlut2 and GluR1, respectively. Relative staining levels of these markers in the glomerular layer were compared. **(B)** A schematic diagram of synapse formation and dendrite selection regulated by Sema7A/PlxnC1 signaling. Sema7A/PlxnC1 signaling is critical for initiating post-synaptic processes in M/T cells. OR-derived neuronal activity in OSNs induces Sema7A expression in OSN axons. Sema7A interacts with its receptor PlxnC1, localized in M/T cell dendrites, to recruit PSD molecules. Synaptic transmission facilitates synaptic competition, driving dendrite selection in M/T cells. Following the formation of synapses between OSN axons and M/T cell dendrites, trans-synaptic activity from OSNs promotes dendrite selection and branch pruning in M/T cells.

To define the olfactory critical period more precisely, experiments involving unilateral naris occlusion were conducted. Occlusion began immediately after birth (P0) and lasted for various durations (Figure 2A). When the occluded naris was reopened before P7, odor sensing and perception remained unaffected. However, occlusions extended beyond P8 significantly delayed synapse formation and dendrite selection (Inoue et al., 2021).

To identify molecules responsible for imprinting, Inoue et al. (2018) analyzed signaling molecules expressed in the neonatal olfactory epithelium and olfactory bulb. Semaphorin 7A (Sema7A) and its receptor Plexin C1 (PlxnC1) were identified as promising candidates. Sema7A, expressed in the axon terminals of OSNs, depends on neural activity, while PlxnC1 is located in M/T cell dendrites, but only during the first week after birth. Knockout (KO) experiments showed that blocking Sema7A/PlxnC1 signaling did not disrupt olfactory map formation, but impaired post-synaptic events and M/T cell dendrite selection (Figure 2B) (Inoue et al., 2018). Additionally, the M/T-cell-specific PlxnC1 conditional KO mice exhibited altered social behaviors later in life, such as avoiding stressful interactions with unfamiliar mice (Inoue et al., 2021).

In gain-of-function experiments, exposing mice to a specific odor during the critical period enlarged the corresponding glomeruli and increased sensitivity to that odor (Valle-Leija et al., 2012; Liu et al., 2016; Inoue et al., 2021). The mice also displayed sustained interest in the odor during habituation/dishabituation tests (Inoue et al., 2021). Stress-induced hyperthermia tests revealed that odor imprinting reduced stress, and notably, this stress-reducing effect was observed even for aversive odors, such as 4-methylthiazole (4MT), a derivative of TMT (Inoue et al., 2021).

What causes the positive quality of imprinted odor memories? Inoue et al. (2021) focused on oxytocin, a hormone crucial for smooth social interactions (Gur et al., 2014; Muscatelli et al., 2018; Bosch and Young, 2018). When oxytocin KO mice were conditioned to 4MT, their sensitivity to the odor increased, but the stress-reducing effect disappeared (Inoue et al., 2021). These mice also failed to acclimate to unfamiliar mice during social-memory tests. Intraperitoneal injections of oxytocin during the neonatal stage rescued impaired social responses. However, these rescue effects were not observed if the injections were administered after the critical period (Inoue et al., 2021). This research suggests oxytocin is necessary during the neonatal stage to impart positive attributes to imprinted memories. Despite this, the precise brain regions where oxytocin acts to encode the odor as positive remain unknown.

Interestingly, neonatal brain development also plays a role in how sensory stimuli are processed. Until around P14, the hypothalamic–pituitary–adrenal (HPA) axis, which regulates stress response, remains underdeveloped (Levine, 1994; Schmidt et al., 2003). As a result, all sensory stimuli, including unpleasant ones, may be perceived as pleasant. Building on this, future studies may explore whether aversive qualities can also be imprinted onto conditioned odors if stress hormones are administered during this critical period.

## Activity-dependent modulation of odor qualities after the critical period

Odor qualities are innately determined, but can be changed by imprinted memory during the neonatal critical period (Inoue et al., 2021; Qiu et al., 2021). Can olfactory imprinting change the texture of an odor even after the critical period?

In rodents, developmental transitions occur during lactation (Figure 1). During the early lactation period (P0-9), pups exhibit an odor preference when paired with a neutral odor and electrical stimulation, facilitated by low stress sensitivity (Schmidt et al., 2003; Rincón-Cortés and Sullivan, 2014). This associative learning changes in the mid-lactation period (P10-15), as stress sensitivity develops and disrupts odor preference learning unless the dam is present to reduce stress. By the late lactation period (P16-weaning), the dam’s stress-reducing effects diminish, and associative learning does not occur even in her presence. Although the physical transition from maternal milk to solid food begins at P17 and concludes around P28 (Ost'adalova and Babicky, 2012), the timing of psychological independence remains unclear.

During lactation, mammalian pups rely on the dam for survival and associative learning related to maternal stimuli. Then, Katori et al. (2025) investigates how maternal preference shifts during the late lactation period in mouse pups. In the study, 4-MT (an aversive odorant)-conditioning during P12-18 was demonstrated to alter pups’ behavior toward 4MT, shifting from avoidance to approach by P19. In the 4MT-conditioned pups, stress hormone release and neuronal activity in key stress-related regions, including the paraventricular nucleus (Kondoh et al., 2016), bed nucleus of the stria terminalis (Somerville et al., 2010; Yassa et al., 2012; Kim et al., 2013), and central amygdala (Davis, 1992; Duvarci and Pare, 2014), were suppressed during 4MT exposure. These results suggest that 4MT-conditioning effectively reduces fear and anxiety responses associated with 4MT exposure. Interestingly, oxytocin receptor knockout did not affect 4MT-preference acquisition, indicating that dam-pup bonding is not essential for this learning (Nagasawa et al., 2012). Arginine vasopressin, a neuropeptide involved in pair-bonding behavior, may be related to the odor-preference acquisition.

Maternal separation during late lactation enhanced odor-preference learning, suggesting that separation becomes positively valued by P17, marking the onset of pups’ psychological independence. The study highlights a sensitive period during mid-lactation for preference acquisition and emphasizes aligning weaning timing with psychological independence to avoid adverse effects (Kikusui et al., 2019). Extended 4MT-nip conditioning showed diminishing acquired preferences by P29, likely coinciding with the end of weaning. Furthermore, sibling interactions during maternal separation were found to modulate stress and learning, as complete separation from both the dam and siblings suppressed odor-preference acquisition. Sibling interactions during maternal separation may play a crucial role in shaping the development of social behaviors post-lactation.

Recent findings indicate that somatostatin-positive neurons in the zona incerta integrate olfactory and somatosensory inputs to establish maternal bonding in pups (Li et al., 2024). These neurons lose their function in adulthood as their projections to other brain regions retract after weaning (Lin et al., 1990; Nicolelis et al., 1995; Li et al., 2024). This developmental shift may underlie the change in the valence of maternal separation, transforming it into a positive experience during late lactation.

Katori et al. (2025) showed that 4MT-conditioning induces both approach behavior and reduced stress responses toward 4MT (Figure 1B). However, the causal relationship between these changes remains unclear. The specific brain regions responsible for approach behavior have not yet been identified. Once identified, causal relationships could be clarified through loss-of-function and gain-of-function experiments.

## Discussion

The concept of critical periods in neural development refers to specific windows of time during which the brain is particularly receptive to environmental stimuli, resulting in enduring changes in neural circuits. While extensively studied in the visual system, the mechanisms underlying critical periods in the olfactory system remain less understood. However, recent research has begun to shed light on the cellular and molecular processes involved in olfactory critical periods, emphasizing their role in shaping neural circuits and behaviors.

The mouse olfactory system presents a unique opportunity for studying critical period plasticity due to its organized glomerular structures in the olfactory bulb (OB). These glomeruli encode specific odor cues during the early stages of processing, providing an excellent model for investigating how environmental stimuli transform into complex behaviors and perceptions through higher brain centers. Several distinct features of this system make it advantageous for understanding the mechanisms of critical periods:

1. Predefined encoding of specific odors: the discrete glomerular structures that encode specific odors are established at the periphery, enabling researchers to trace morphological, physiological, and behavioral changes across different individuals.
2. Interaction of genetic programs and environmental factors: the critical period for olfaction coincides with postnatal development of the immature olfactory circuit, during which environmental factors influence genetically programmed circuit development. For example, dendrite selection by OSN axons in the mouse OB is modulated by Sema7A/PlxnC1 signaling (Inoue et al., 2018; Inoue et al., 2021). This interaction provides a valuable framework for understanding how external stimuli shape brain circuits during development.
3. Common features with other sensory systems: studies have revealed that the olfactory critical period shares key characteristics with critical periods in other sensory systems, including defined time windows, dependency on sensory input onset, and high structural and functional plasticity affecting adult behavior and perception.

For future research, defining the critical period more precisely will be essential. Previous studies on the olfactory critical period have primarily focused on the axonal projections of OSNs to the OB during a plastic window, where neural activity shapes the olfactory map. However, if the critical period is defined differently, behavioral changes induced by odor exposure may not alter the olfactory map formed by OSNs but instead affect higher brain regions that regulate behavior. These regions could influence OB plasticity through top-down mechanisms. For example, functional feedback loops exist between the mouse OB, piriform cortex, and basal forebrain (Shipley and Adamek, 1984). Additionally, sex differences between males and females should be considered. Investigating cortical feedback mechanisms—enhancing odor discrimination, identity, and coding—will offer valuable insights into the dynamic coordination of neural circuits. Moreover, integrating experience-dependent glomerular circuitry development with behavioral state-dependent OB modulation can serve as a powerful model for understanding complex neural interactions during critical periods.
